# The Role of the Chaplain as a Patient Navigator and Advocate for Patients in the Intensive Care Unit: One Academic Medical Center’s Experience

**DOI:** 10.1007/s10943-019-00865-z

**Published:** 2019-06-22

**Authors:** Paula Teague, Susan Kraeuter, Sarah York, Wayman Scott, Muhammad M. Furqan, Sammy Zakaria

**Affiliations:** 1 Department of Spiritual Care and Chaplaincy, Johns Hopkins University, Baltimore, MD USA; 2 Johns Hopkins Bayview Medical Center, 4940 Eastern Avenue, Baltimore, MD 21224 USA; 3 Department of Medicine, Johns Hopkins University School of Medicine, Baltimore, MD USA; 4 Johns Hopkins University School of Nursing, Baltimore, MD USA; 5 Department of Pastoral Counseling, Loyola University Maryland, Baltimore, MD USA; 6 Department of Cardiovascular Medicine, Heart and Vascular Institute, Cleveland Clinic, Cleveland, OH USA

**Keywords:** Patient-centered care, Patient navigation, Patient advocacy, Chaplain, Spiritual care, Intensive care

## Abstract

Effective communication between intensive care unit (ICU) staff, and patients and their families, can help increase understanding, trust, and goals-of-care decisions. Many strategies focus on enhancing communication by increasing family meetings or adding patient navigators. In our ICU, we implemented both strategies, uniquely appointing a chaplain for the patient navigator role. We then surveyed ICU staff on their perceptions of the chaplain/patient navigator, which yielded several valuable insights. Although all staff supported a strong chaplaincy presence, many had concerns about the dual chaplain/patient navigator role. Based on our mixed results, we encourage further exploration to optimize the chaplain role in the ICU.

## Introduction

The intensive care unit (ICU) environment is a stressful environment for patients, families, and the medical team (Henrich et al. 2016; van Mol et al. 2015). Most patients who are admitted into the ICU are critically ill, with many eventually dying or leaving with temporary or permanent disabilities. Our cardiac and medical ICUs are not an exception, and we have witnessed many difficult situations.

Notably, we cared for a young woman a few years ago who had a respiratory arrest following presumed heroin use. Although she eventually was stabilized, she required a long period of cardiopulmonary resuscitation, needed many medications to restore circulation, and needed mechanical ventilation. At no time was she responsive and brain imaging revealed impending brain herniation which is always associated with impending death. Within hours, ICU nurses and physicians noted several significant social issues. Importantly, her family had strongly held conflicting spiritual beliefs and systems of faith, which affected their approach to medical care and made coping and decision-making more difficult.

As a result, there were significant conflicts between the patient’s husband and parents, as well as her extended family. Eventually, it led to conflicts between the patient’s family and with ICU staff in addition to interpersonal conflicts between ICU staff. Some family members perceived the ICU staff to be disorganized and disinterested, while others viewed communication between staff and family members as intermittent and inadequate to address conflicting family views. ICU staff needed several prolonged family meetings over many days before they regained rapport and trust with the upset family, which fortunately occurred before the patient’s death.

This event crystalized the need for a coordinated approach to family meetings in which ICU team members work together with patients and families to freely communicate on goals of care throughout the patient’s stay in the ICU. This required an interdisciplinary approach, necessitating close coordination between nurses, physicians, social workers, chaplains, and other hospital staff members.

## Background

Two interventions were subsequently designed to offer intensive family support. The first introduced the concept of regular and frequent family meetings, with an initial mandatory meeting held within 24 h, a larger interdisciplinary meeting held within 3 days of admission, and subsequent meetings held at least weekly. The second intervention assigned ICU patients a patient navigator (Shelton et al. 2010). Uniquely, we utilized a patient navigator with a chaplaincy background, who also received special training into the dynamics of setting up and running a family meeting. Based on Darnell’s description of the responsibilities of a patient navigator (which are listed in Table 1) (Darnell 2007), we felt that a chaplain could effectively function in this role, because chaplains are typically adept at facilitating interpersonal communication, have some knowledge of community organizations that can offer patients and families support, and can also assist in overcoming barriers preventing optimal recovery and health. Chaplains are already familiar with the care needs for patients in the ICU (Fitchett 2017), serving as a liaison/consultant with the medical team (Carey and Cohen 2009), managing emotional and spiritual pain, participating in end-of-life decision-making (both in and out of the ICU setting) (Lee et al. 2018), addressing “do not resuscitate” and withdrawal of life-support decisions, and dealing with bioethical issues (Timmins et al. 2017). This combination of attributes and roles of chaplains is clearly important and underutilized (Choi et al. 2015).

**Table 1:** Patient navigator roles (Darnell 2007)

Patient navigator role	Project interpretation	Chaplain skill set
1. Act as a contact	Assist in coordination of family meetings to increase communication among staff, patient, and family members	Professional chaplains are trained to recognize gaps in communication and facilitate management of these gaps. This includes mediating personal relationships as well as healthcare conversations where the patient needs an advocate to assist in achieving clarity
2. Involve community organizations	The patient navigator can connect the patient and family to community-based resources, including faith-based communities	Chaplains have connections to community faith groups including congregations and other service-based associations. These medical-religious partnerships are sometimes critical to supporting health as social agency-based connections
3. Introduce and determine patient eligibility for clinical trials	Not applicable. Our intervention did not address this aspect of patient navigation	Chaplains can help build trust with religious congregations and leaders, which enable discussion of research in a context of transparency and true consent. However, our patient navigator was not asked to introduce clinical trials with patients or family members, since no trial needed assistance enrolling patients within the ICUs during the intervention time period
4. Anticipating, identifying, and helping patients overcome barriers within the healthcare system	Patient navigators could help address barriers that might exist in order to assist patients to receive proper care	Chaplains working with medical–religious partners can identify barriers to healthcare and address them in the congregational setting
5. Coordinating with health insurance ombudsman	Not applicable. Our intervention did not address this aspect of patient navigation	This is not part of the chaplain skill set. Social workers and case managers are better suited to address issues with health insurance
6. Coordinating ongoing outreach to health disparity populations	Goals-of-care meetings to enhance communication and understanding can help achieve health equity. In addition, the patient navigator is empowered to meet with patients and family members at any time to ensure understanding of medical issues and facilitate good decision-making	Chaplains are strongly aware of the importance of social justice and can help both identify and address health disparities. Many have identified congregational medical/preventive health issues that are then addressed through programming in the congregational setting

For the purpose of understanding the chaplain resident role in our ICUs, the model for providing spiritual care services relies upon an accredited clinical pastoral education (CPE) program led by a certified educator working with up to eight CPE residents who provide much of the direct clinical services. There are also two staff chaplains who function as mentors and liaisons with the clinical units so that the CPE residents have additional educators and mentors. For this intervention, the chaplain resident assigned to the intervention already had completed one unit of CPE and was specifically assigned a CPE educator and a clinical mentor who were familiar with the project. Because the CPE resident did not have the experience of a certified chaplain, additional ad hoc oversight was offered to the CPE resident providing spiritual care.

In this paper, we mainly focus on the role of the chaplain as a patient navigator, discussing the views of ICU staff and our interpretation of the chaplain’s effectiveness in this unique role.

## Methods

This study was conducted in the 12-bed Cardiac ICU and the 12-bed Medical ICU at the Johns Hopkins Bayview Medical Center. The Johns Hopkins Institutional Review Board acknowledged this study as a “quality improvement” project rather than “human subjects research” (Study #:NA_00071963) and exempted the study from further review. Prior to any interventions, ICU staff participated in 1-h online and in-person training modules teaching principles of interdisciplinary communication, family meetings, and addressing the special needs of patients and families. In addition, a 1-h online training module was developed to specifically introduce the novel chaplain/patient navigator role. These training modules were developed by our research team. For the attending medical staff, the study interventions were introduced and discussed in multiple faculty meetings. For the chaplain/patient navigator, who was also a CPE resident, 8 h of individual training sessions were conducted by our research program coordinator and project leaders.

The study interventions were then introduced in March 2015 and continued until December 2015 for a total of 10 months. During this time period, nursing staff attended 1-h monthly voluntary workshops to further advance the role of the chaplain/patient navigator. In addition, rotating residents and fellows attended biweekly 30-min training sessions organized by the study investigators, who taught how to conduct successful multidisciplinary family meetings and introduced the chaplain/patient navigator role to the trainee physicians. Lastly, the chaplain/patient navigator and research program coordinator met weekly for 1-h educational and feedback sessions.

During the intervention time period, patients admitted into either ICU were randomly assigned by a computerized random-number generator to the “usual care” group or to the intervention group coordinated by the chaplain/patient navigator. For patients in the intervention group, ICU staff were required to attend frequent multidisciplinary family meetings, as described in the introduction. In addition, a chaplain/patient navigator was assigned to each patient. In our study, we also asked our chaplain/patient navigator to be primarily responsible for setting up and attending all family meetings, which would increase the visibility of the chaplain/patient navigator to patients, families, and ICU staff.

### Data Sources and Interpretation

At the end of the intervention time period, we asked for qualitative and quantitative data by surveying ICU nurses and medical trainees about their perceptions of the chaplain/patient navigator. We did not include surveys from chaplains, physical therapists, occupational therapists, and social workers, because there were comparatively few (*N* = 5) who worked in the ICU setting and were not part of the research team. For the nurses and physicians, individual invitations were sent through REDCap (Harris et al. 2019), an online-only, electronically secure, and anonymous data-capture instrument. If an invitation was not addressed, three additional reminder emails were sent weekly. All responses were anonymous. Staff and trainees were invited to respond to five specific questions (listed in Table 2) related to the patient navigator role and were also asked to offer additional comments. For three (Questions 1, 2, and 3) of the questions, respondents were asked to choose one of five answers (strongly agree, agree, neutral, disagree, or strongly disagree). Question 4 required a Yes/No response, and Question 5 required respondents to free text an answer. At the end of the survey, each respondent was also encouraged to offer additional qualitative comments about the intervention. After the survey closed, we collated and downloaded all responses into a Excel (Microsoft Office, 2016) spreadsheet for analysis and interpretation. Of note, we further obtained additional qualitative data separate from the survey by collecting anecdotes in ad hoc interviews with ICU staff.

**Table 2:** Patient navigator survey questions

1.	The patient navigator was useful in gathering health care team and family members for family meetings
2.	The patient navigator increased collaboration between staff members
3.	The patient navigator was helpful liaison between the patient/family and health care team
4.	Is a chaplain training and background appropriate for the patient navigator role?
5.	What profession is most suited for the patient navigator role?

### Statistical Analysis

No pre-specified statistical analyses were performed since this was an exploratory study in anticipation of further investigations and interventions. However, we performed a post hoc descriptive analysis of the response rates, gender categories, clinician types, and level of training (if applicable). A Chi-square test was performed comparing the frequencies of responses for Questions 1–4 by physicians and nurses. Pearson Chi-square values were calculated, and Phi and Cramer’s *V* tests were performed when applicable to measure the strength of association. For the Chi-square test, all the responses were categorized into a physician group (combining attendings, fellows, residents, and interns) and a nursing group. To simplify statistical analysis, we combined the Likert scale categories of strongly agree and agree and strongly disagree and disagree into two categories and retained neutral responses in the third category. All the analyses were performed using SPSS Statistics 24 (IBM Corp., 2016) software.

## Results

We emailed 283 individual survey invitations to physicians and nurses and received 152 responses for a 54% response rate. Table 3 shows the respondent numbers, gender distribution, clinician type, and physician level of training. Nurses accounted for 24% of responses, with physicians comprising the remaining 76%. Approximately half (51%) of all respondents were women. As shown in Table 4, a majority of the respondents felt that the chaplain/patient navigator was useful in gathering individuals for family meetings, with physicians more inclined to agree [72% vs. 48%, *χ*^2^ (2, *N* = 152) = 8.280, *p* = 0.016]. In addition, a majority of physicians (especially the trainee physicians) agreed that the chaplain/patient navigator increased collaboration; however, the nurses were less supportive [58% vs. 38%, *χ*^2^ (2, *N* = 151) = 6.196, *p* = 0.045]. Similarly, physicians generally agreed that the chaplain/patient navigator was a helpful liaison between the patient/family and the healthcare team, while nurses were less supportive [64% vs. 35%, *χ*^2^ (2, *N* = 149) = 9.523, *p* < 0.01]. Viewpoints on the appropriateness of chaplain training/background for the patient navigator roles were most divergent, with the physicians more favorable. In contrast, a majority of nurses thought the chaplain background was not appropriate for the patient navigator role [80% vs. 42%, (*χ*^2^ (1, *N* = 149) = 17.143, *p* < 0.001, *Ø* = 0.356, *p* < 0.001]. In summary, physicians were much more favorable to the chaplain/patient navigator role compared to nurses.

**Table 3:** Training level and gender distribution of the survey respondents

Clinicians	Female *N* = 78 (51%)	Male *N* = 74 (49%)	Total *N* = 152
Nurses	31 (84%)	6 (16%)	37
Physicians	47 (41%)	68 (59%)	115
Attendings	6 (43%)	8 (57%)	14
Fellows	7 (35%)	13 (65%)	20
Residents	27 (43%)	36 (57%)	63
Interns	7 (39%)	11 (61%)	18

**Table 4:** Responses to survey questions by profession

Responses	Profession				
	Attendings (%)	Fellows (%)	Residents (%)	Interns (%)	Nurses (%)
Q1: The patient navigator was useful in gathering health care team and family members for family meetings					
Agree	64	85	68	78	48
Neutral	29	0	26	17	30
Disagree	7	15	6	5	22
Q2: The patient navigator increased collaboration between staff members					
Agree	43	70	58	56	38
Neutral	43	10	32	33	35
Disagree	14	20	10	11	27
Q3: The patient navigator was a helpful liaison between the patient/family and health care team					
Agree	72	70	63	56	35
Neutral	14	5	27	33	41
Disagree	14	25	10	11	24
Q4: Is a chaplain training and background appropriate for the patient navigator role?					
Yes	71	95	79	71	42
No	29	5	21	29	58
Q5: What profession is most suited for the patient navigator role?					
Physician	14	17	19	24	37
Nurse	7	11	16	6	23
Chaplain	0	17	11	24	14
Social worker	71	44	45	47	23
Case assistant	7	11	8	0	3
Other	0	0	0	0	0

Physicians = attendings, fellows, residents and interns; nurses = registered nurses

Also, there were varied opinions about which profession was most appropriate for the patient navigator role. For physicians, a plurality favored social worker training; however, some thought physicians or nurses would be most appropriate. For the nurses, a plurality favored physicians to take the patient navigator role, followed by nurses or social workers. Some respondents thought the chaplain role was most appropriate for the patient navigator role, including trainee physicians (fellows 17%, residents 11%, interns 24%) and nurses (14%).

In addition to the numerical responses to questions, 63 respondents included additional comments, with 11 specifically addressing the role of the chaplain as the patient navigator. Many specifically commented positively on a chaplain’s ability to facilitate family meetings and communication: “*Primarily non*-*clinical*, *can help translate conversation*.” (Fellow 1) “*Chaplain and social worker are often detached from the actual administration of medical care and therefore serve as a valuable liaison if communication between patients and families is lost*.” (Resident 1) “*A Chaplain is a neutral person*, *non*-*medical personnel but still familiar with and comfortable with the hospital and our policies*.” (Intern 1) “*They have more free time and they are trained to be in tune with other aspects of the patient’s lives outside of their medical needs*.” (*Nurse 6*) “*Families trust religious staff and feels comfortable talking with them*.” (*Nurse 7*)

Other comments seemed to value the role of the chaplain in offering support and welcomed including chaplains in multidisciplinary family meetings; however, there were some comments that were unhappy with the chaplain’s responsibility to set up family meetings, including: “*Have a Chaplain as part of the team but not as navigator*,” (*Nurse 3*) and “*There were times when meetings were just not a good time to do and felt like the navigator did not understand that the nurse is busy and cannot always be a part of a meeting even though I understand a nurse is a crucial part to being involved*,”(*Nurse 5*) and “*Having the patient navigator be conscious and respectful of everyone’s time*. *Having family meetings are important however*, *it does not come as a priority in urgent situations*. *An example*, *the patient navigator interrupting during a critical situation to make the nurse aware that a family meeting has been scheduled*.” (*Nurse 1*)

In addition, there were some comments that questioned the role of the patient navigator/chaplain, including the following: “*When families introduced to Chaplain they did not know why a Chaplain was involved*, *especially if their spiritual needs did not fit with a chaplain*.” Other comments had difficulty in reconciling their views to the “*Chaplain*-*supportive role*.”

In some comments, respondents felt that the chaplain/patient navigator should have more training in the medical sphere, commenting: “*They have no medical background and often times provides families with totally incorrect information*.”(*Nurse 4*) “*When I reviewed his assessments from meetings I attended*, *I often found his interpretations of the families’ statements about their wishes and decisions to be different from mine*. *I feel this often came from inadequate understanding of the medical details*.” (*Attending 1*) “*Additional training may be useful including being provided a ‘script’ to approach families*.” (Nurse 8)

In addition to the survey data results, we collected three anecdotes highlighting the difficulties encountered by the chaplain as a patient navigator. The first centered on the chaplain/patient navigator wanting to contact a family member without clarifying the legal relationship hierarchy in surrogate patient decision-making. The second focused on a family with a highly religious value system who did not want the chaplain as a navigator but rather with a sole focus as their spiritual care provider. The third referred to a family frightened by the chaplain’s call to set up a family meeting, because family members thought chaplain contact meant that something was seriously wrong with their loved one.

## Discussion/Lessons Learned

It is clear that chaplain/patient navigators are useful, especially in setting up and participating in family meetings. However, there are mixed views on whether chaplains functioning in the patient navigator role is ideal. Most view chaplains as helpful in increasing collaboration and acting as a patient-staff liaison; nevertheless, many feel that other professions were better suited for this role. Based on our experience, we believe successful implementation of chaplains in this role is difficult and may prevent widespread use despite the theoretical qualifications of chaplains that make them uniquely qualified to assume the role of a patient navigator (Furqan and Zakaria 2017). Based on our quantitative and qualitative results, there are several issues listed in Table 5 that must be addressed, which are described below.

1. The dual chaplain/patient navigator role is a double-edged sword.

Using chaplains as patient navigators simultaneously offers advantages and disadvantages. ICU staff recognize chaplains’ unique skill sets, which make them well qualified to set up and facilitate family meetings. However, by virtue of the chaplain’s background, the medical team, patients, and family members then expect spiritual care services, which preclude performing the time-consuming role of a patient navigator. In many cases, families asked for prayer and other religious resources, and did not focus on the logistics needed for a successful family meeting, despite attempts to refer spiritual care needs to another chaplain. In one situation, a family complained about their experience with the “reluctant” chaplain, who they perceived was not whole-heartedly invested in caring for them spirituality. In addition, a few of the initial interactions with the chaplain patient navigator were fraught with anxiety, distress, and distrust, because chaplains are often associated with end-of-life or crisis situations or overtly religious concerns.

**Table 5:** Lessons learned

1.	The dual chaplain/patient navigator role is a double-edged sword
2.	It is imperative to clarify roles and expectations
3.	It is critical to have all ICU staff “buy in” to the concept of a chaplain/patient navigator
4.	Including chaplains in family meetings is beneficial
5.	Interprofessional education in the ICU is important and needed
6.	Consistent feedback and supervision of the chaplain/patient navigator is required
7.	Introduce the chaplain/patient navigator into the ICU without including additional interventions

There was also confusion with the medical team about the new combined chaplain/patient navigator role. Chaplains have always been a hospital team member; however, taking the lead to establish family meetings was novel. Nurses and physicians struggled to fulfill chaplain requests to attend family meetings. In some situations, nurses were frustrated with the chaplain when asked for their attendance when they had to address urgent patient responsibilities. In the new role of patient navigator, the chaplain needed to push for ICU staff attendance and was perceived to be occasionally unsupportive, unaware of competing responsibilities, and in a few cases, impolite. In retrospect, assigning a more experienced chaplain may have helped. In addition, more ICU staff education about the novel chaplain/patient navigator role would have been useful. Finally, frequent regular check-ins with ICU staff may have addressed other negative perceptions.

2. It is imperative to clarify roles and expectations.

Chaplains/patient navigators need to operate in two professional spheres. They need to perform spiritual assessments as well as set up and facilitate goals-of-care meetings. However, most chaplains do not have formalized medical training—although admittedly some have a background in nursing or allied health training. In our experience, the chaplain was often asked to address medical issues, and it was rather difficult to defer those questions and decisions to the medical team. Upon reflection, several ICU staff echoed the idea that the chaplain may have been more effective with more medical education or training in social work, or with provision of mutually agreed-upon goals and expectations, including the use of standardized scripts. If chaplains are to be responsible for family meetings, we agree on the need for greater didactic and experiential training in interacting with medical staff, and also agree on the need for providing clear expectations on their role in discussing medical issues when interacting with patients and families.

3. It is critical to have all ICU staff “buy in” to the concept of a chaplain/patient navigator.

Interdisciplinary collaborations are most successful, both in terms of process and outcomes, when there is enthusiasm and a deep commitment to the project (Dedhia et al. 2008). However, many ICU staff did not support the idea of a chaplain/patient navigator and did not value the paramount importance of frequent family meetings. Nurses frequently highlighted the need to balance attending family meetings with competing demands, and understandably did not like to be interrupted when they were acutely caring for critically ill patients. As a result, the chaplain/patient navigator role was associated with a problematic overall intervention and ICU staff, especially the nurses, were much more unfavorable to the idea of a chaplain serving as a patient navigator. Therefore, it is critical to spend the time and effort to exclusively introduce the chaplain’s new role.

In addition, many physicians were not appropriately informed about the chaplain/patient navigator and the responsibility to attend frequent family meetings. In our academic ICUs, attendings, fellows, residents, and interns frequently rotated on-and-off service. Every week, new physicians arrived with varied levels of knowledge about the interventions, had varied investment in the new model, and needed to learn about several other topics as well. Although we led biweekly educational sessions that introduced the new role of the chaplain to trainees and addressed the challenges of running multidisciplinary family meetings, the overall “buy in” and interest in family meetings was not uniform. Based on our experience, we believe that any novel intervention of this type needs more frequent stakeholder education and motivation.

4. Including chaplains in family meetings is beneficial.

Chaplain involvement is beneficial for patients in the ICU settings, particularly for those at the end of life (Balboni et al. 2013; Choi et al. 2015; Ernecoff et al. 2015). Hospital-based chaplain counseling may lead to more referrals to hospice and palliative care and fewer deaths in the ICU. However, many ICUs, including ours, do not routinely ask chaplains to attend multidisciplinary family meetings. Our interventions clearly allowed for more chaplain involvement, which was recognized as valuable. In many family meetings, the topics discussed were often distressing, and chaplains were helpful in offering support and spiritual guidance to patients and family members. Thus, no respondent favored exclusion of chaplains from family meetings and would continue to welcome chaplains. However, some ICU staff did not favor having chaplains in the role of patient navigator. Based on our experience, we agree that these two roles should be separate, unless the chaplain is provided with intensive training and has the support of almost all the ICU staff. Otherwise, other means of assuring the chaplain’s attendance at the family meetings are required.

5. Interprofessional education in the ICU is important and needed.

The very nature of ICU work requires a great deal of interdisciplinary collaboration and communication. Our intervention to increase effective family meetings and introduce the role of the chaplain/patient navigator helped to increase collaboration. However, our intervention did not have a strong interprofessional education (IPE) component, which would have helped to increase familiarity with the project, and helped various disciplines work better. As an example, many ICU staff do not know the qualifications and skill sets of chaplains (Best et al. 2016). Likewise, chaplains are not familiar with the roles and nature of the roles of others on the medical team. We believe that better implementation of an interdisciplinary project requires IPE, which can help identify assumptions about professional roles and either affirm or clarify these assessments. In addition, IPE experiential practice and simulation training can help identify and address potential pitfalls and allow participants to become more invested in the interventions. Finally, IPE can help introduce ICU staff to novel concepts, especially if the chaplain is to perform tasks that are outside of the normal scope of a chaplain’s function.

6. Consistent feedback and supervision of the chaplain/patient navigator is required.

We used a chaplain resident as our patient navigator/chaplain. While the chaplain had multiple mentors and was also supervised by a certified CPE educator, we believe that even more feedback and intensive supervision is needed for a new chaplain to succeed in this role. In addition, the chaplain’s supervisors and mentors also needed more education to optimally guide the chaplain/patient navigator, as they were also actively engaged in learning about this new role. In reflection, consistent multidisciplinary supervision of the project is needed, which could consist of weekly meetings with an experienced physician, nurse, social worker, and chaplain. We strongly believe that increased feedback is required for ensuring the success of a chaplain/patient navigator, since any potential pitfalls can be proactively identified and addressed. By having multiple disciplines offer feedback and oversight, there can be greater investment and “buy in” into the intervention goals.

7. Introduce the chaplain/patient navigator into the ICU without including additional interventions.

In this intervention, we simultaneously introduced a chaplain/patient navigator into the ICU along with implementing a program to increase regular multidisciplinary meetings. Although we believed that this is reasonable, in retrospect it would have been better to introduce the chaplain/patient navigator into the ICU without any specific responsibilities to organize or hold family meetings. In this manner, ICU staff can become more familiar with the chaplain as a patient advocate and offer feedback and input on optimizing the chaplain’s role. Once addressed, the chaplain then has goodwill that can engender support for taking on additional responsibility to arrange and facilitate family meetings.

## Conclusions

In the ICU, emphasizing goals to increase communication and collaboration can help increase patient, family (Furqan and Zakaria 2017; Kynoch et al. 2016; Sadler et al. 2014; Stricker et al. 2009), and staff satisfaction (Blake et al. 2013; Manojlovich and DeCicco 2007). There is growing recognition of the role of chaplains in healthcare (Fitchett 2017; Lee et al. 2018; Newell and Carey 2000; Saad and de Medeiros 2016), and it is a worthwhile goal to expand the traditional role of the chaplain as a comforter and mediator. Our intervention was planned to address these goals and was universally recognized by ICU staff to have good intentions. However, we encountered challenges, which may have limited the effectiveness of the chaplain in the patient navigator role. We learned several important lessons, and encourage future studies utilizing chaplain/patient navigators that build on our interventions. Based on our experiences, we certainly agree that the chaplain should have an expanded role, since chaplains are great advocates and sources of support for patients and families. However, we are warier of having chaplains serve as the patient navigator. We have mixed opinions on whether they should be in this novel role, or whether individuals from other backgrounds, such as nursing, social work, or other allied health professions (Browne et al. 2015), could be more effective. However, we all agree that there is a need for a strong and ongoing presence for chaplaincy in the ICU to ensure that spiritual care interventions (e.g., assessments, support, counseling, education, ritual and worship activities) are fulfilled for the benefit of patients and their families, and staff.
